# Transcranial magnetic stimulation and transcranial direct current stimulation: treatments for cognitive and neuropsychiatric symptoms in the neurodegenerative dementias?

**DOI:** 10.1186/s13195-014-0074-1

**Published:** 2014-11-10

**Authors:** Greg J Elder, John-Paul Taylor

**Affiliations:** 1 Institute of Neuroscience, Newcastle University, Campus for Ageing and Vitality, Newcastle upon Tyne NE4 5PL, UK

## Abstract

**Introduction:**

Two methods of non-invasive brain stimulation, transcranial magnetic stimulation (TMS) and transcranial direct current stimulation (tDCS), have demonstrable positive effects on cognition and can ameliorate neuropsychiatric symptoms such as depression. Less is known about the efficacy of these approaches in common neurodegenerative diseases. In this review, we evaluate the effects of TMS and tDCS upon cognitive and neuropsychiatric symptoms in the major dementias, including Alzheimer’s disease (AD), vascular dementia (VaD), dementia with Lewy bodies (DLB), Parkinson’s disease with dementia (PDD), and frontotemporal dementia (FTD), as well as the potential pre-dementia states of Mild Cognitive Impairment (MCI) and Parkinson’s disease (PD).

**Methods:**

PubMed (until 7 February 2014) and PsycINFO (from 1967 to January Week 3 2014) databases were searched in a semi-systematic manner in order to identify relevant treatment studies. A total of 762 studies were identified and 32 studies (18 in the dementias and 14 in PD populations) were included.

**Results:**

No studies were identified in patients with PDD, FTD or VaD. Of the dementias, 13 studies were conducted in patients with AD, one in DLB, and four in MCI. A total of 16 of the 18 studies showed improvements in at least one cognitive or neuropsychiatric outcome measure. Cognitive or neuropsychiatric improvements were observed in 12 of the 14 studies conducted in patients with PD.

**Conclusions:**

Both TMS and tDCS may have potential as interventions for the treatment of symptoms associated with dementia and PD. These results are promising; however, available data were limited, particularly within VaD, PDD and FTD, and major challenges exist in order to maximise the efficacy and clinical utility of both techniques. In particular, stimulation parameters vary considerably between studies and are likely to subsequently impact upon treatment efficacy.

## Introduction

Dementia is associated with significant financial and societal costs. The worldwide cost of dementia in 2009 was estimated to be US$422 billion [1]. By 2050, the number of new cases is projected to be more than double the incidence in 2000 [2]. Individuals with dementia display a range of associated cognitive and neuropsychiatric sequelae. Whilst a number of medications are used to manage these symptoms, pharmacological treatments have only a limited degree of efficacy and in some cases (for example, antipsychotics) may be accompanied by significant side effects. Therefore, there is an urgent need to develop alternative treatments.

One area which has garnered considerable clinical and research interest recently is the use of non-invasive brain stimulation. In this review, we explore whether two of the most common of these approaches, transcranial magnetic stimulation (TMS) and transcranial direct current stimulation (tDCS), might be used to treat symptoms associated with the most common forms of neurodegenerative disease, including Alzheimer’s disease (AD), vascular dementia (VaD), dementia with Lewy bodies (DLB), Parkinson’s disease with dementia (PDD) and frontotemporal dementia (FTD). Additionally, we consider the application of TMS and tDCS in people with mild cognitive impairment (MCI), which is a recognised risk state for the development of dementia and, in particular, dementia associated with AD [3]. Similarly, we also consider the application of both techniques in individuals with Parkinson’s disease (PD), as PD is considered to be a significant risk factor for PDD [4] and cognitive deficits are apparent even in early PD [5].

### Methods of non-invasive stimulation

#### Transcranial magnetic stimulation

TMS modulates cortical plasticity, where a brief (100 μs) electrical current is delivered through a coiled wire placed on the scalp, resulting in a time-varying magnetic field across the skull (1.5 to 2 Tesla), which induces an electric field and subsequently alters neuronal activity [6],[7]. The dosage is typically determined by the stimulation intensity and is often calibrated to the person-specific motor-evoked potential (MEP) threshold [8]. Whilst safe, the most common side effect of TMS is transient pain (with a prevalence of approximately 40%), typically dependent on an individual’s tolerability to the location, intensity or frequency of stimulation [7]. TMS cannot be used in individuals with metallic equipment situated near the coil and carries a low risk (<1% in normal populations) of inducing seizures [7]. Stimulation protocols include ‘single-pulse’ stimulation, ‘paired-pulse’ TMS and repetitive TMS (rTMS); in the latter, low-frequency (≤1 Hz), high-frequency trains, or varying bursts of stimulation (for example, theta-burst stimulation (TBS)) can be delivered [7].

Cortical neurons 1.5 to 3 cm below the scalp may be activated, depending on the stimulation intensity. Increasing the stimulation intensity and frequency increases cortical disruption; however, stimulation trains modulate cortical excitability, and the subsequent effects depend on the stimulation parameters. Typically, higher frequencies (for example, 20 Hz) increase and lower frequencies (approximately 1 Hz) suppress cortical excitability [6],[7],[9], although low-frequency stimulation may not always cause inhibitory effects [10]. Continuous TBS results in inhibitory after-effects, whilst intermittent TBS results in facilitatory after-effects, at least in the case of the motor cortex [11]. The duration of after-effects are affected by the stimulation protocol. Single-pulse and short rTMS protocols result in after-effects lasting a few seconds; long or multiple rTMS trains result in after-effects of several minutes to 1 hour; and TBS protocols can result in after-effects of up to 8 hours [12],[13]. Longer effect durations can occur with TMS, when repeated daily, as poststimulation effects can be observed even up to 1 month later [14].

TMS has been shown to reduce neuropsychiatric symptoms and improve cognition. In older people, for example, a double-blind study in adults over 50 years of age with memory problems showed that rTMS led to improvements in associative memory [15] and an open-label study showed that rTMS over a 3-week period significantly reduced the severity of depressive symptoms in older adults [16]. These data are supported by meta-analytic results demonstrating that high-frequency TMS applied to the left dorsolateral prefrontal cortex (DLPFC) was superior to sham stimulation in double-blind treatment studies of depression, with effects comparable to pharmacological treatment [17]. Importantly, the therapeutic effects of TMS may be additive, as repeated sessions over a 6-week period have been suggested to lead to cumulative improvements in mood [18]. TMS may also modulate and enhance motor learning, vision, memory and attention in healthy individuals [13]. Taken together, these results suggest that TMS might be a potential method of treatment for cognitive and neuropsychiatric symptoms associated with dementia.

#### Transcranial direct current stimulation

Another non-invasive treatment method is tDCS, where a weak electrical current is delivered through two scalp electrodes by a portable battery-powered stimulator. An anodal and cathodal electrode, typically between 25 cm^2^ and 35 cm^2^ in size, are inserted into holding bags moistened with saline or conductive gel and are placed on the scalp in accordance with the International 10–20 system [19]. The current density, calculated on the basis of the power intensity divided by the area of the electrode, is used as a marker of dosage and influences the after-effects [19]. Current densities of 0.05 mA/cm^2^ are typical, although they can range from 0.02 mA/cm^2^ to 0.08 mA/cm^2^[19]. It has been speculated that tDCS modulates spontaneous neuronal activity in a polarity-specific manner [20], whereby the tDCS current has a modulatory effect upon cortical excitability by either increasing or decreasing intrinsic neural firing. Specifically, anodal stimulation typically increases the membrane potential by several millivolts, whereas cathodal stimulation typically results in an opposite effect, decreasing the membrane potential [19],[21],[22]. However, these effects do not appear to be consistent across studies [19],[23]. There are no reports of serious adverse effects with tDCS; common side effects include mild tingling, fatigue and light itching under the electrodes [19].

Relevant normative and clinical studies suggest that tDCS may be a useful therapeutic tool. For example, it may have utility in the treatment of depression; in one study individuals who received active tDCS showed an improvement in mood compared to placebo stimulation followed by an open-label phase [24]. Accompanying short-term improvements in attention and working memory were also observed [24]. Post-tDCS improvements have also been shown in young healthy controls in terms of visuomotor coordination [25] and working memory performance [26]. Similar to TMS, the benefits of tDCS may be additive. In one study, participants who had received active tDCS over 5 consecutive days displayed greater performance on a motor skill task compared to sham stimulation, with these effects persisting at a 3-month follow-up time point [27].

## Methods

### Search methods and inclusion and exclusion criteria

In order to evaluate the clinical utility of either TMS or tDCS in the symptomatic treatment of AD, VaD, DLB, PD, PDD, FTD or MCI, only treatment studies were included. PubMed (until 7 February 2014) and PsycINFO (from 1967 to January week 3, 2014) databases were searched using the following terms: “Alzheimer*”, “dementia”, “frontotemporal dementia”, “vascular dementia”, “Parkinson*”, “Parkinson’s disease with dementia”, “Lewy body*”, “cognitive impairment” AND “magnetic stimulation” or “current stimulation”. As this review was focussed on the treatment of cognitive and neuropsychiatric symptoms, studies which used either TMS or tDCS to specifically ameliorate other symptoms alone (for example, motor symptoms) were excluded.

**Figure 1:**
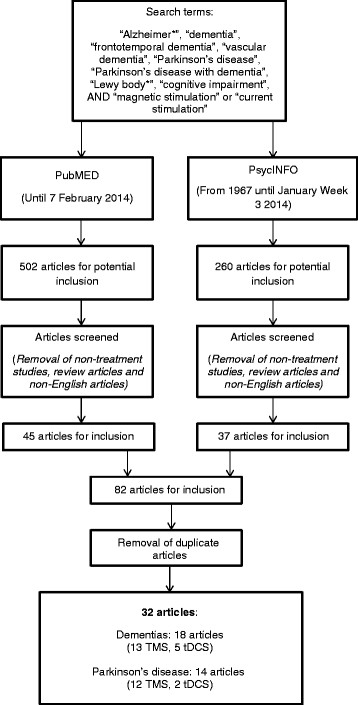
Study search strategy and selection process.

This search strategy (Figure 1) resulted in 762 potential articles for inclusion. The examination of relevant review papers did not result in any additional articles for inclusion. Article titles and abstracts were examined and non-treatment studies, review, non-English and duplicate articles were removed, leaving a total of 32 studies (18 within the dementias and 14 in PD populations).

## Results

The majority of the studies in the dementias examined cognitive outcome measures [28]-[42] whilst several examined neuropsychiatric symptoms [43]-[45]. A total of 13 studies targeted AD patients [28]-[33],[37]-[42], 4 studies included MCI patients [34]-[36],[43] and 1 study involved DLB patients [44]. No studies reported the therapeutic use of TMS or tDCS in individuals with VaD, PDD, or FTD. A total of 14 studies examined cognitive and neuropsychiatric outcome measures in individuals with PD [46]-[59]. Within dementia, improvements were shown on at least 1 measure in 16 of the 18 studies, and TMS was the most common method of stimulation (13 studies). In the dementias, improvements were shown in cognitive (Additional file 1: Table S1) and neuropsychiatric symptom domains (Additional file 2: Table S2). The variety of symptoms and outcomes reported precluded any formal statistical meta-analysis. In PD, cognitive and neuropsychiatric improvements were observed in 12 of the 14 studies (Additional file 3: Table S3), and TMS was the most common modality, being used in a total of 12 studies.

## Discussion

### Treatments targeting cognition in Alzheimer’s disease

In AD, there was a general trend for improvements across a wide range of cognitive outcome measures following treatment with TMS and tDCS [28]-[42]. Typical sites of stimulation included the DLPFC [28]-[31],[37],[42], temporal regions [38], temporoparietal regions [41] or a combination of multiple regions [32],[33],[39]. However, sample sizes were often small (including single-case studies [36],[37],[43]), and the majority were open-label in design. Nevertheless, these studies will help inform future work because several concepts, which are likely to impact upon the utility and effectiveness of non-invasive stimulation, were apparent:

1. The frequency of stimulation is an important factor in the field of neurostimulation in general and is therefore very likely to influence any potential cognitive improvements that might occur in dementia. In one study, Mini Mental State Examination (MMSE), Instrumental Activities of Daily Living Scale (IADL) and Geriatric Depression Scale (GDS) scores were shown to improve in patients with AD following high-frequency rTMS, but not following low-frequency or sham stimulation [28].

2. The benefits of TMS or tDCS may also be highly task-specific, as, for example, AD patients showed improvements within an action-naming task compared to sham TMS, but not within an object-naming task [29]. Boggio and colleagues showed that active tDCS resulted in improvements in visual recognition memory, but not in selective attention or working memory performance [39]. Similarly, in another study, improved visual recognition performance was shown compared to sham tDCS; however, cognitive (MMSE & Alzheimer's Disease Assessment Scale - cognitive subscale (ADAS-Cog)) and visual attention measures were unaffected [38].

3. There was some evidence to suggest that the beneficial effects of TMS could be sustained in AD, with one study reporting that auditory sentence comprehension improvements were observed two weeks after stimulation, although this benefit was not sustained across various other cognitive measures, including MMSE scores [31]. Preliminary findings in another study in AD with tDCS have shown that 4 weeks of active stimulation led to MMSE, action-naming task and noun-naming task improvements at a 12-week follow-up time point, although the stimulation parameters were not reported in this study, making replication challenging [40].

4. Dementia severity may affect the TMS response, as whilst poststimulation action-naming and object-naming improvements have been reported in patients with moderate to severe AD compared to sham stimulation, only action-naming improvements have been observed in patients with mild AD [30]. In contrast, in another study with a crossover design in patients with MCI, the effects of TMS upon a range of neuropsychological tests, including executive function, attention, working memory, psychomotor speed and visuomotor coordination, were negligible [35], thus making it difficult to conclude whether the level of cognitive impairment (or lack thereof) is important for TMS response or nonresponse.

5. The location of stimulation is likely to have a major influence upon the therapeutic efficacy of TMS or tDCS. For example, low-frequency rTMS delivered to the right DLPFC resulted in improvements in non-verbal recognition task performance in MCI patients, compared to left DLPFC or sham stimulation [34]. For tDCS, current polarity is likely to be an important factor for treatment efficacy; one study reported that whilst word recognition improvements in an AD cohort were shown after bilateral anodal stimulation, no improvements were observed following sham stimulation and performance worsened following cathodal stimulation, with visual attention unaffected [41].

6. Other studies have combined TMS with cognitive training. This has a neurobiological basis, as rTMS has been suggested to influence learning in a neuroplastic fashion, with consequent changes to synaptic function [12]. In patients with AD, Bentwich and colleagues trialled cognitive tasks, whose operation was ‘localised’ to specific brain regions (Broca’s area, Wernicke’s area, left and right DLPFC and left and right parietal somatosensory association cortex), including syntax, grammar, action-naming and spatial memory tasks, with the difficulty adjusted on the basis of patient performance [32]. These brain regions were targeted using rTMS whilst the cognitive tasks were performed. Poststimulation improvements were shown in the primary outcome measure (ADAS-Cog), but not on the secondary outcome measure (MMSE). However, that study was hampered by the small sample size (*n* = 8), the lack of a placebo condition and the inability to separate the effects of TMS from the effects of cognitive training. In a similar study, Rabey *et al*. assessed the effects of combined rTMS and cognitive training on ADAS-Cog scores (primary measure) in patients with AD [33], but included a placebo rTMS and cognitive training group. Compared to the placebo condition, positive results were observed at two follow-up time points (6 weeks and approximately 4 months). However, similar to the study conducted by Bentwich and colleagues, it is unclear whether rTMS or cognitive training alone resulted in the beneficial effects, or whether the effects were combined.

Overall, it is evident that there is a great deal of methodological heterogeneity in the use of non-invasive brain stimulation in these studies. In order to advance the use of both techniques, the replication of studies is necessary.

### Treatments targeting cognition in other dementias and mild cognitive impairment

We found no studies in which researchers examined the cognitive benefits of either TMS or tDCS in VaD, DLB, PDD or FTD. Four studies have examined the effects of TMS upon individuals with MCI, although, we found no studies using tDCS in this group. One cross-over study showed that TMS resulted in only negligible effects upon a range of neuropsychological tests in patients with MCI (*n* = 7) [35] and a further study showed that TMS resulted in an improvement in non-verbal recognition memory [34]. A single-case study suggested that TMS resulted in improvements to a range of measures, including associative memory [36].

### Treatments targeting cognition and neuropsychiatric symptoms in Parkinson’s disease

The majority of studies conducted in individuals with PD employed TMS (12 of the 14 studies; Additional file 3: Table S3) and improvements were observed in 12 of the studies. As in the dementias, a great deal of methodological heterogeneity was apparent; however, the overall results suggest that TMS might benefit depressive symptoms in patients with PD and that the positive effects may persist at follow-up time periods of up to 8 weeks in some cases [47],[48],[50]-[52],[54],[56],[57]. Nevertheless, the ability to directly translate these positive findings to dementia patient groups might be limited, as the investigators in these studies did not knowingly include, or report findings on, PD patients with cognitive impairment. As was the case in studies conducted in other populations, an obvious limitation is the open-label nature of several studies [51],[52],[56]. In all cases, adequately powered, placebo-controlled studies are necessary to confirm these promising results.

Several trials incorporated treatment and placebo combinations of anti-depressant medication and TMS, generally showing comparable improvements in mood to those observed using anti-depressant medication with sham TMS [48],[50],[53]. However, due to the lack of a combined active TMS and anti-depressant group, it has not been possible to examine whether or not these effects are additive.

The effects of TMS upon cognition are less clear, as improvements in a range of secondary outcome measures have been reported in individuals with PD, including neuropsychological tests [48],[55],[57] and MMSE scores [50], although these findings are not consistent across all studies [58]. However, the effects of TMS might be location-specific, since in one crossover study Tower of London task improvements were observed after rTMS was delivered to the right DLPFC, and not the left DLPFC [59].

Only two studies to date have employed tDCS within PD [46],[49]; one of which observed that 2 mA of anodal stimulation applied to the prefrontal cortices did not improve depression, quality of life, or measures within a reaction time task [46]. However, Boggio and colleagues observed that 2 mA, but not 1 mA, of anodal stimulation applied to the left DLPFC resulted in improved accuracy on a working memory task [49], suggesting that the current density might be an important factor in maximising the efficacy of techniques. Benninger *et al*. [46] did not observe any effects of stimulation upon measures of depression or quality of life, or within a reaction time task. However, in this randomised, double-blind, sham-controlled study the aim was to examine whether tDCS benefitted motor symptoms, and there were no differences shown between active and sham stimulation. It is possible that the lack of studies which have used tDCS within PD or PDD for cognitive symptoms is due to the preoccupation of its effect (or absence thereof) upon motor symptoms [60]; nevertheless, the use of tDCS in order to examine any non-motor benefits should be encouraged.

### Treatment targeting neuropsychiatric symptoms in dementia

Very few studies have specifically examined the effects of TMS or tDCS upon neuropsychiatric symptoms in dementia, either as a primary or a secondary outcome measure. The limited published data has primarily reported on TMS and its use as a treatment of depression; for example, TMS has been reported to reduce depressive symptoms in suspected or probable DLB patients with treatment-resistant depression [44]. However, a major limitation of this particular study was the lack of a placebo group and the small sample size (*n* = 6) [44]. Another small study reported improvements in depressive symptoms in AD patients, although this was not a primary outcome measure [28]. Perhaps the most rigorous study examining the use of non-invasive stimulation in the treatment of neuropsychiatric symptoms was a double-blind, sham-controlled study which found that tDCS was not an effective method in the treatment of apathy within AD, with no benefit shown upon secondary outcomes including depression, cognition or other neuropsychiatric symptoms [45].

Whilst there is a substantive evidence base for the use of TMS in the treatment of psychosis in schizophrenia [61], we did not find any studies where TMS or tDCS was used to treat psychosis in dementia. Notably, a single-case study conducted in an MCI patient reported a reduction in the frequency of auditory verbal hallucinations following TMS, which was accompanied by a reduction in threatening content during the hallucinations and in associated distress [43].

### Challenges of using transcranial magnetic stimulation and transcranial direct current stimulation in dementia

Overall, there is a clear dearth of high-quality and adequately-powered trial data regarding the use of TMS and tDCS in the treatment of cognitive and neuropsychiatric symptoms in dementia, although the findings for both stimulation modalities are generally suggestive of a potential therapeutic benefit. The lack of robust data may reflect the relative novelty of these approaches and their use in dementia, and also the practical difficulties of performing intervention studies in these patient groups.

Additionally, as is typical for new modalities of treatment, and due to the partial neurobiological understanding of how both methods modulate cognitive and neuropsychiatric symptoms, a significant number of challenges remain for the optimisation of the therapeutic benefits:

1. Dementia patients are frequently on a range of psychotropic medications, and it is well-established that firstly, a wide array of drugs can interact with the effects of TMS, and secondly, TMS can itself affect neurotransmitters and neuromodulators [62]. Thus, psychotropic medication use, in conjunction with non-invasive stimulation, might potentially lead to unexpected effects, by either enhancing or suppressing any treatment benefits arising from TMS or tDCS. For example, in the case of tDCS, administration of the *N*-methyl-D-aspartate (NMDA) receptor antagonist dextromethorphan has been shown to suppress the effects of anodal and cathodal tDCS [63], which may have implications for the concurrent use of memantine, an NMDA receptor antagonist which is used as a symptomatic treatment in dementia [64]. Examples of other psychotropic drugs which are sometimes prescribed for dementia and could potentially interact with tDCS include carbamazepine, citalopram, amphetamine, levodopa and lorazepam, amongst others [20],[63].

2. From a stimulation perspective, the treatment response in patients with dementia could also be affected by changes in brain morphology. Structural brain lesions can affect the tDCS current flow [20], and TMS is highly dependent upon the distance between scalp and cortical surface [65]. Grey matter atrophy can alter the effect of TMS, as the cortical current density is dependent upon the degree of atrophy [66]. This is particularly relevant to dementia populations, since extensive atrophy, particularly in AD [67],[68], might also affect the treatment response. There are techniques which can potentially overcome this difficulty, including, for example, calibrating the distance of the TMS coil to each individual patient in accordance with the degree of atrophy [69]. In the case of tDCS, computational techniques can potentially model the current flow within atrophied brains and thereby assist in the optimisation of electrode montages, or electrode design, within the dementias [70],[71]. It is also possible that these techniques could ultimately be used to model the response to stimulation [70] at an individual patient level; therefore, the clinical utility of such techniques should be examined.

3. Furthermore, the effects of stimulation, and specifically the TMS current flow, may also be affected by other factors, including the distribution of cerebrospinal fluid (CSF), which has an amplifying effect due to the increased CSF conductivity compared to other brain tissue [72]. Therefore, ventricular enlargement, which is a common feature in neurodegenerative diseases, could be influential in determining the stimulation intensity needed to obtain a treatment effect.

4. A further challenge is that treatment studies must clearly define the specific symptom targeted by TMS or tDCS, which is likely to depend on the population. For example, individuals with AD are likely to present with primarily amnestic deficits (particularly in the early stages) whereas other dementias such as DLB or PDD may display various symptoms including cognitive fluctuations, and attentional or visuoperceptual dysfunction [73]. Moreover, where a target symptom has been clearly defined, an appropriate clinical measure for assessing the treatment response to TMS/tDCS is needed. This can be particularly difficult for neuropsychiatric symptoms where scales of individualised symptoms often lack good reliability and validity and are not sensitive to the symptom change over time [74].

5. Determining the most appropriate location for TMS or tDCS will complicate any trial design, although aetiological models of a particular symptom may aid the choice of location. For example, such models have implicated the underactivity of DLPFC in depression [75], and thus non-invasive stimulation over this area may be the most appropriate location for treating depression. Nevertheless, the effects of TMS and tDCS may not be limited to one particular area and are likely to modulate activity in other regions [76],[77]. Therefore, models suggesting localised cortical effects under the TMS coil or tDCS electrodes are potentially over-simplistic and more research is needed to understand the overall network effects of stimulation.

6. Perhaps one of the greatest challenges lies in the wide range of TMS and tDCS stimulation parameters which could be (and indeed have been) applied in dementia populations (Additional file 1: Table S1 and Additional file 2: Table S2). Thus, further work is needed to determine the optimal stimulation parameters for treatment. This includes the current density (in the case of tDCS) and the stimulation frequency, the number of treatment sessions and how often they occur, as well as the duration, and the interval between stimulation sessions, since these factors are likely to significantly impact upon stimulation efficacy. In particular, maximising the after-effects will be particularly important if non-invasive treatments are to be clinically viable in dementia populations, and help minimise the number of treatment sessions needed to obtain meaningful symptom improvements. For example, in the case of TMS, the use of TBS protocols might maximise any after-effects [12],[13], although the utility and safety of this approach in dementia populations has yet to be elucidated.

Overall, these factors prevent definitive conclusions being made about the underlying true efficacy of non-invasive stimulation in the treatment of dementia symptoms, with possible false negatives arising as a consequence of small sample sizes and suboptimal stimulation. Conversely, as the majority of reported studies are open-label or uncontrolled in their design, reports will be inevitably biased towards positive outcomes; therefore, caution needs to be exercised in reaching definitive conclusions about efficacy. Negative results have been reported in the use of tDCS [78]. Importantly, in order to avoid potential positive publication biases, and advance the clinical utility of both techniques, it is strongly recommended that researchers fully report all trial results, including negative findings. This approach will undoubtedly help to define the most appropriate stimulation parameters needed to obtain a given cognitive effect.

There are a variety of ways in which the challenges outlined above may be overcome. Trials in healthy individuals, or in those with mild disease, may allow finessing of stimulation parameters and establish the tolerability of protocols. It should be recognised that there may be a direct interaction between pathology and the subsequent treatment response, which may make the response more or less likely in milder disease [30]. Therefore, normative studies, in conjunction with either single-case or small open-label patient studies, would be helpful in establishing treatment parameters and elucidating the potential efficacy of TMS and tDCS. Positive outcomes in these studies might then inform larger trials with double-blind, placebo-controlled designs which can lend themselves to meta-analytic approaches. Issues with adequate blinding and the lack of good placebos have bedeviled therapeutic trials with TMS [79]. In this respect, tDCS may offer an advantage over TMS, as double-blind placebo designs are much more robust using tDCS [80]. That said, there are difficulties in conducting double-blind studies using both tDCS and TMS, although possible solutions exist (such as increasing the ramping-up time within tDCS studies) [81].

The efficacy of treatment might also be maximised through methods of stratification, where patients are selected on the basis of, for example, neuropsychological performance, genetics or physiological markers. Various studies have adopted this approach. Where probable AD patients were stratified on the basis of MMSE performance, mild patients showed improved post-rTMS action-naming task performance, whilst moderate-to-severe patients showed improved action-naming and object-naming task performance [30]. Similarly, post-rTMS improvements in depression and cognition have been shown in mild-to-moderate, but not severe, AD patients [28].

From a genetic perspective, Brunoni and colleagues demonstrated that the 5-HTTLPR serotonin transporter polymorphism predicted the treatment response to tDCS treatment for depression, where long/long homozygotes displayed a larger improvement compared to short allele carriers, in a dose–response manner [82]. This is also relevant to TMS, as plasticity has been shown to be affected by the brain-derived neurotrophic factor (BDNF) Val66Met genotype [12]. Obvious polymorphic targets in dementia could include apolipoprotein E (APOE) and microtubule-associated protein τ (MAPT), given their known association with AD and α-synuclein related disorders [83]. Specifically in PD, dysexecutive impairments and activity in frontoparietal executive networks may depend on functional polymorphisms in the dopamine regulating the catechol-*O*-methyltransferase (COMT) enzyme [84]. Hypothetically, stratification on the basis of the COMT allelic expression in these patients and those with PDD might determine treatment responsivity. Finally, physiological predictors or biomarkers could be considered; in an rTMS study, the cerebral blood flow of patients with treatment-resistant major depressive disorder was assessed pre-treatment and post-treatment [85]. Individuals who showed a response to treatment showed greater baseline levels of resting state blood flow at the rTMS site compared to individuals who did not respond to treatment.

Other methodological factors might increase stimulation efficacy. Techniques to aid TMS coil placement may improve the treatment response, such as stereotactic systems which can enable specific regions to be targeted on the basis of an individual patient’s structural magnetic resonance imaging (MRI) scan. The use of MRI may therefore reduce the number of patients required, optimise stimulation intensities needed to overcome potential changes in brain morphology, and increase effect sizes in a TMS study, owing to the increased spatial accuracy offered by this individually-targeted approach to stimulation [8]. In the case of tDCS, the size and shape of the electrode may affect the treatment response by altering the focality of the current [19]. Most tDCS studies use two rectangular electrodes for stimulation. However, methods such as high-definition tDCS, where four cathode electrodes are positioned around an anodal electrode, may allow for better regional targeting [71]. The therapeutic effects of alternating polarity during repeated stimulation sessions of tDCS, where, for instance, anodal stimulation could be applied before cathodal stimulation, are yet to be explored, but as polarity-dependent effects have been observed within tDCS [19], this approach may be worthy of further investigation.

The relative advantages and disadvantages of both techniques should be taken into consideration. However, TMS and tDCS cannot be directly compared due to the differences in their mechanisms of action. Both techniques cause different physiological effects, as TMS can directly elicit action potentials, which is not the case with tDCS [76],[86]. Furthermore, TMS can also affect areas which are distant, yet functionally connected, to the stimulated region [87]. Importantly, as a technique, tDCS is a neuromodulator rather than a method of extrinsic stimulation, and is therefore dependent on the pre-existing neural state. This may have implications in the dementias, where intrinsic neural states are potentially different from healthy brains; therefore the translation of tDCS effects from healthy controls to patient populations should be done with caution.

A further advantage of TMS is that the manipulation of timing parameters can result in varied cortical effects [10]-[13], which could potentially be used to maximise treatment efficacy. However, TMS is a more expensive method than tDCS, needs a degree of technical expertise to deliver and the magnetic coil is required to be held still during stimulation, which may be challenging in cognitively-impaired and often physically frail patient groups. The use of coil holding rigs and/or robotic arm systems may help to overcome this issue [88]. Currently, an advantage of TMS over tDCS is the greater focality of stimulation [80].

In contrast, there are advantages in the use of tDCS, as tDCS stimulators are typically inexpensive, battery-operated and extremely portable. Compared to TMS, tDCS might also have safety advantages, since no serious adverse effects have been reported as a result of this technique [19]. Moreover, since the placement of tDCS electrodes follows the standard International 10–20 system [89], it is feasible to train non-specialists and/or carers to cheaply administer tDCS to patients either within a home or clinical environment. However, as more studies are conducted within dementia, it is likely that the choice of technique will ultimately depend upon the level of efficacy in treating specific symptoms.

## Conclusions

TMS and tDCS may have potential as interventions for the treatment of symptoms associated with dementia. However, there are very limited available data in the use of these approaches in the symptomatic treatment of the dementias, the majority of trials contained inadequate control arms, and no data has been reported in several major dementia groups (e.g. VaD, PDD, and FTD). Even in studies with positive outcomes, effect sizes have been small and the clinical significance of these remains to be established. Major challenges exist in terms of appropriate patient selection and optimisation of the stimulation parameters to obtain an efficacious response. However, if these issues can be surmounted, non-invasive stimulation might provide a novel and alternative therapeutic paradigm for symptom management in dementia.

## Abbreviations

AD: Alzheimer’s disease

ADAS-Cog: Alzheimer’s Disease Assessment Scale–Cognitive

APOE: Apolipoprotein E

BDNF: Brain-derived neurotrophic factor

COMT: Catechol-*O*-methyltransferase

CSF: Cerebrospinal fluid

DLB: Dementia with Lewy bodies

DLPFC: Dorsolateral prefrontal cortex

FTD: Frontotemporal dementia

IADL: Instrumental Activities of Daily Living Scale

MAPT: Microtubule-associated protein tau

MEP: Motor-evoked potential

MCI: Mild cognitive impairment

MMSE: Mini Mental State Examination

MRI: Magnetic resonance imaging

NMDA: *N*-methyl-D-aspartate

PD: Parkinson’s disease

PDD: Parkinson’s disease with dementia

rTMS: Repetitive transcranial magnetic stimulation

TBS: theta-burst stimulation

tDCS: Transcranial direct current stimulation

TMS: Transcranial magnetic stimulation

VaD: Vascular dementia

## Competing interests

The authors declare that they have no competing interests.

## Authors’ contributions

GJE and JPT conceived the review, and drafted and edited the manuscript. GJE conducted the literature searches. GJE and JPT read and approved the final manuscript.

## Additional files

## Supplementary Material

Additional file 1: Table S1. Noninvasive stimulation studies targeting cognition in dementia. Click here for file

Additional file 2: Table S2. Noninvasive stimulation studies targeting neuropsychiatric symptoms in dementia. Click here for file

Additional file 3: Table S3. Noninvasive stimulation studies targeting neuropsychiatric symptoms in Parkinson’s disease. Click here for file
