# Supplementary material for: Transcranial magnetic stimulation and transcranial direct current stimulation: treatments for cognitive and neuropsychiatric symptoms in the neurodegenerative dementias?
Source: Alzheimers Res Ther. 2014 Nov 10;6(9):74. doi: 10.1186/s13195-014-0074-1 (PMC4255638; doi:10.1186/s13195-014-0074-1)
Supplement: Additional file 1: Table S1. — Noninvasive stimulation studies targeting cognition in dementia. [file s13195-014-0074-1-S1.docx]

| Additional Table 1:  *Non-invasive stimulation studies targeting cognition in dementia* | | | | | |
| --- | --- | --- | --- | --- | --- |
| Study | Patient group | Treatment | Stimulation parameters | Outcome measures | Results |
| Ahmed *et al.* (2012) [28] | Probable AD (*n* = 45; NINCDS-ARDA criteria) | TMS | rTMS applied over right DLPFC before left DLPFC (approx. 10mins per hemisphere): 2 treatment groups (20Hz rTMS @ 90% MT or 1Hz rTMS @ 100% MT) and 1 sham group. | GDS, MMSE and IADL. | GDS (↓3.3 points), MMSE (↑4.2 points), and IADL (↑4.6 points), improved at 3-month follow-up after 20Hz rTMS, in mild/moderate AD but not severe AD. |
| Bentwich *et al*. (2011) [32] | Probable AD (*n* = 8) | TMS | 10Hz rTMS applied to Broca’s area, left and right DLPFC (90% MT), Wernicke’s area, left and right pSAC (110% MT), with three areas stimulated per day during treatment phase (1 session per day, 5 days a week for 6 weeks) and during maintenance phase (2 sessions per week for 12 weeks). Various cognitive tasks included with patient-specific levels of difficulty. | Primary outcome measures: ADAS-Cog, CGIC  Secondary outcome measures: ADAS-ADL, HAM-D, MMSE, NPI. | Improvements shown in ADAS-ADL (↑4.9 points at 6-week follow-up) and ADAS-cog (↓4.2 points and ↓4 points at 6 weeks and 4.5 months follow-up). |
| Boggio *et al*. (2009) [39] | AD (*n* = 10; NINCDS-ARDA criteria with MMSE of 12-25) | tDCS | Active (2mA for 30 minutes) or sham (2mA for 30 seconds) stimulation (35cm² electrodes). Anode placed on left DLPFC (F3) or left temporal cortex (T7) with cathode on right supraorbital area.  Left DLPFC, left temporal cortex and sham stimulation given in counterbalanced manner with 48 hours washout period between each condition. | Selective attention (Stroop), working memory (backward and forward digit span) and visual recognition memory tasks. | Improvement shown in visual recognition memory task after active stimulation (approx.18% improvement after temporal and 14% after prefrontal stimulation)  compared to sham. |
| Boggio *et al*. (2012) [38] | AD patients (*n* = 15; NINCDS-ADRDA and DSM-IV criteria, no other neuropsychiatric diseases and MMSE >15). | tDCS | Active or sham anodal stimulation (2mA for 30 mins) delivered to T3 and T4 bilaterally (35cm² electrodes with 64cm² electrode placed over right deltoid muscle) for 5 consecutive days. | MMSE, ADAS-Cog, visual recognition and visual attention tasks. | Improvement shown on visual recognition task compared to baseline after active stimulation, with improvements sustained at 1 month follow-up period (11.4% improvement). No effects shown on MMSE, ADAS-cog or visual attention. |
| Cotelli *et al*. (2006) [29] | Probable AD (*n* = 15; NINCDS-ADRDA criteria). | TMS | Left DLPFC (approx. F3/F4), right DLPFC (approx. F7/F8) or sham 20Hz rTMS (90% RMT). 10 × 600ms pulses given from visual stimulus onset during task. | Action-object picture naming task. | Action naming improved following left and right DLPFC stimulation compared with sham (15-20% improvement), with no effects shown in object naming. |
| Cotelli *et al*. (2008) [30] | Probable AD (*n* = 24; NINCDS-ADRDA criteria). | TMS | Left DLPFC (approx. F3/F4), right DLPFC (approx. F7/F8) or sham 20Hz rTMS (90% RMT). 500ms pulses given from visual stimulus onset during task. | Action-object picture naming task. | Mild patients showed improved action naming (approx. ↑20%) performance following DLPFC stimulation compared with sham. Moderate to severe patients showed improved action (approx. ↑15%) and object naming (approx. ↑10-15%) performance DLPFC stimulation compared to sham. |
| Cotelli *et al*. (2010) [40] | AD patients (*n* = 14) | tDCS | 4-week active tDCS (unspecified treatment parameters) or 2-week sham tDCS followed by 2-week active stimulation. | MMSE, unspecified neuropsychological and action-object naming tasks. | Incomplete pilot study: improvements in MMSE, action naming and noun-naming tests at 12-week follow-up (changes in MMSE and tests not specified). |
| Cotelli *et al*. (2011) [31] | Probable moderate AD (*n* = 10; NINCDS-ADRDA criteria) | TMS | Group 1: 4-week active 20Hz rTMS (100% MT) applied to left DLPFC, with one 25min session for five consecutive days.  Group 2: 2-week sham rTMS followed by 2-week active stimulation (identical stimulation parameters to Group 1). | Neuropsychological tests (memory, executive function and language). | Improvements shown in auditory sentence comprehension (Group 1 only at 2-week follow-up; ↑10.7 points) and in  auditory sentence comprehension at 12 weeks post-treatment, irrespective of group (↑10.9 points). |
| Cotelli *et al*. (2012) [36] | Single case study: 61-year-old man with amnestic MCI | TMS | rTMS (20Hz, 100% MT) applied to left IPL for 25 mins a day, 5 days a week for 2 weeks during treatment phase. | Associative memory (face-name) task and battery of neuropsychological assessments. | Improved face-name association task performance between baseline and 2 week follow-up period (approx. 15-20% improvement). Improvement stable at 24-week follow-up although lower than performance of healthy controls. Improvements shown in auditory-verbal learning and primacy neuropsychological test 2-weeks post TMS (on basis of test cut-off scores), but only improvement in primacy test remained at 24 weeks (↑4 points) |
| Devi *et al*. (2014) [42] | Possible or probable AD (*n* = 12); NINCDS-ADRDA criteria, with evidence of aphasia | TMS | 4 sessions over 2-week period (90% MT applied to both sides of DLPFC: 10Hz for first 6 patients and 15Hz for second 6 participants)  rTMS (100% output to DLPFC for 30 mins) over 2 weeks at baseline. | Subtests of BDAE (nonverbal agility, verbal agility, complex ideational material, naming, responsive naming and commands), category fluency and MMSE. | Improvements in BDAE nonverbal (↑1.9 points) and verbal agility (↑1 point) at 4-week follow-up time point. |
| Ferrucci *et al*. (2008) [41] | Probable AD (*n* = 10; NINCDS-ADRDA and DSM-IV criteria, with MMSE > 20). | tDCS | Stimulation (1.5mA for 15 mins; 0.06mA/cm² current density) with one electrode placed over scalp and reference over right deltoid muscle. Anodal, cathodal or sham tDCS delivered bilaterally to tempoparietal areas (P3-T5 on left side and P6-T4 over right side) in a random order with a minimum 1-week interval between stimulation. | Word recognition and visual attention tasks. | Word recognition improved after anodal tDCS (↑2.4 correct responses), remained the same after sham stimulation and worsened after cathodal stimulation (↓2.6 correct responses; at 30 minutes post-tDCS compared to baseline). No effects observed on visual attention task. |
| Haffen *et al*. (2012) [37] | Single case study: 75-year-old man with probable AD (NINCDS-ADRDA criteria). | TMS | 10Hz rTMS administered to left DLPFC (100% MT) during 10 stimulation sessions (20 mins) over 2 weeks. | Neuropsychological tests (including MMSE) and visuospatial function, episodic memory and information processing tests. | Improvements were shown in 8 out of 10 tests 1 month post-TMS. Greatest improvements were shown in episodic memory (↑160% free recall scores and ↑133% memory impairment scores compared to baseline) |
| Rabey *et al*. (2013) [33] | Probable mild to moderate AD (*n* = 15; DSM-IV criteria). | TMS | 10Hz rTMS (active/sham) applied to left and right DLPFC (90% MT), Broca’s area (90% MT), Wernicke’s area (110% MT) and left and right pSAC (110% MT). Broca’s area, Wernicke’s area and right DLPFC treated on Days 1, 3 & 5, with other areas treated on Days 2 and 4. Patients were treated with intensive (1 session per day, 5 days per week for 6 weeks) and maintenance (2 sessions per week for 3 months) phases of treatment. | Primary outcome measure: ADAS-Cog  Secondary outcome measures: CGIC, NPI. | ADAS-cog improvements were shown between baseline and 6 weeks follow-up (↓3.76 points) and between baseline and 4.5 months follow-up (↓3.52 points) compared to sham, alongside improvements in CGIC. |
| Sedlackova *et al*. (2008) [35] | MCI of the vascular type without dementia (*n* = 7; DSM-IV criteria and MMSE ≥ 24). | TMS | Two sessions of 10Hz rTMS 100% MT) over left DLPFC and MC (control site) and two sessions of 1Hz rTMS (100% MT) over same sites, using crossover study design. | Neuropsychological tests assessing executive function and attention, working memory, psychomotor speed and visuomotor co-ordination. | No effects upon any measure. |
| Turriziani *et al*. (2012) [34] | MCI patients  (*n* = 8). | TMS | 1Hz rTMS (90% MT) delivered to left and right DLPFC. Active or sham stimulation spaced 6hrs apart. | Non-verbal recognition memory tasks (face and building recognition). | Improvement in non-verbal recognition performance (↑approx 10% compared to sham) after active stimulation to right DLPFC. |
| Abbreviations: AD: Alzheimer’s disease, ADAS-Cog: Alzheimer Disease Assessment Scale – cognitive, BDAE: Boston Diagnostic Aphasia Examination, CGIC: Clinical Global Impression of Change scale, DLB: dementia with Lewy Bodies, DLFPC: dorsolateral prefrontal cortex, DSM-IV: Diagnostic and Statistical Manual of Mental Disorders, 4^th^ Edition, GDS: Geriatric Depression Scale, HAM-D Hamilton Rating Scale for Depression, Hz: hertz, IADL: Instrumental Activities of Daily Living Scale, IPL: inferior partietal lobules, MC: motor cortex, MCI: Mild Cognitive Impairment, MT: motor threshold, MMSE: Mini Mental State Examination, NINCDS-ADRDA: National Institute of Neurological and Communicative Disorders and Stroke Alzheimer’s Disease and Related Disorders Association, NPI: Neuropsychiatric Inventory, rTMS: repetitive transcranial magnetic stimulation, pSAC: parietal somatosensory association cortex, tDCS: transcranial direct current stimulation, TMS: transcranial magnetic stimulation. | | | | | |
