# Supplementary material for: Transcranial magnetic stimulation and transcranial direct current stimulation: treatments for cognitive and neuropsychiatric symptoms in the neurodegenerative dementias?
Source: Alzheimers Res Ther. 2014 Nov 10;6(9):74. doi: 10.1186/s13195-014-0074-1 (PMC4255638; doi:10.1186/s13195-014-0074-1)
Supplement: Additional file 2: Table S2. — Noninvasive stimulation studies targeting neuropsychiatric symptoms in dementia. [file s13195-014-0074-1-S2.docx]

| Additional Table 2:  *Non-invasive stimulation studies targeting neuropsychiatric symptoms in dementia* | | | | | |
| --- | --- | --- | --- | --- | --- |
| Study | Patient group | Treatment | Stimulation parameters | Outcome measures | Results |
| Marras & Pallanti (2013) [43] | Single case study: 76-year-old woman with dementia and auditory hallucinations. | TMS | 10Hz rTMS to tempoparietial cortex (80% MT) over 15 consecutive days. | Auditory verbal hallucinations. | Reduced frequency of hallucinations following treatment and a reduction in threatening content. Reductions also shown in associated distress. |
| Suemoto *et al*. (2014) [45] | Moderate or probable AD (*n* = 40; NINCDS-ARDA criteria) with MMSE score of 10-20. | tDCS | Active (2mA for 20 minutes) or sham (2mA for 20 seconds) stimulation (35cm² electrodes). Anode placed on left DLPFC with cathode placed above right orbit, with 6 sessions over 2-week period. | Primary outcome: Apathy Scale.  Secondary outcomes:  NPI, caregiver burden, cognition (ADAS-Cog), depressive symptoms, tDCS-related adverse events. | No effects upon primary or secondary outcome measures. |
| Takahashi *et al*. (2009) [44] | DLB patients (*n* = 6), 5 with suspected DLB and 1 with probable DLB. | TMS | 10Hz rTMS delivered to left DLPFC (100% MT). 1Hz stimulation delivered to right DLPFC (110% MT) Treatment repeated for 10 days. | Depressive symptoms (HAM-D scores). | Significant improvement in depressive symptoms (↓13 points - HAM-D score) following rTMS. |
| Abbreviations: AD: Alzheimer’s disease, DLB: dementia with Lewy Bodies, DLFPC: dorsolateral prefrontal cortex, HAM-D Hamilton Rating Scale for Depression, Hz: hertz , MT: motor threshold, MMSE: Mini Mental State Examination, NINCDS-ADRDA: National Institute of Neurological and Communicative Disorders and Stroke Alzheimer’s Disease and Related Disorders Association, rTMS: repetitive transcranial magnetic stimulation  tDCS: transcranial direct current stimulation, TMS: transcranial magnetic stimulation. | | | | | |
