# Supplementary material for: Transcranial magnetic stimulation and transcranial direct current stimulation: treatments for cognitive and neuropsychiatric symptoms in the neurodegenerative dementias?
Source: Alzheimers Res Ther. 2014 Nov 10;6(9):74. doi: 10.1186/s13195-014-0074-1 (PMC4255638; doi:10.1186/s13195-014-0074-1)
Supplement: Additional file 3: Table S3. — Noninvasive stimulation studies targeting neuropsychiatric symptoms in Parkinson’s disease. [file s13195-014-0074-1-S3.docx]

| Additional Table 3:  *Non-invasive stimulation studies targeting neuropsychiatric symptoms in Parkinson’s disease* | | | | | |
| --- | --- | --- | --- | --- | --- |
| Study | Patient group | Treatment | Stimulation parameters | Outcome measures | Results |
| Benninger  *et al.* (2010) [46] | PD (*n* = 25) | tDCS | Active or sham stimulation. Anode placed over premotor and motor (centre of electrode 10mm anterior to Cz) or prefrontal cortices (placed on the forehead above eyebrows). Cathodes placed on mastoids. Target area alternated (total of four periods of stimulation per area).  Stimulation (2mA) delivered for 20 minutes in eight sessions over 2.5 weeks. Sham condition placed small (9cm^2^) anode and cathode electrodes over forehead for 1-2 minutes. | BDI, SF-12v2, SRTT (in addition to motor primary outcome measures). | No significant changes in BDI, SF-12v2 or SRTT. |
| Benninger  *et al.* (2011)  [47] | PD (*n* = 26) | TMS | Randomised controlled trial: iTBS (real or sham) delivered in 8 sessions over 2 consecutive weeks (2 × 1 session per day for 4 consecutive days per week). iTBS parameters: 3 pulses (80% AMT) at 50Hz, repeated at 200ms (5 Hz) intervals for 2 seconds (10 bursts). Trains were repeated 20 times every 2 seconds. iTBS applied to primary motor (M1) and DLPFC areas. | BDI, SF-12v2, SRTT, (in addition to motor  primary outcome measures). | Improvements in BDI in active group (↓4.3 points 1 day after final intervention and ↓2.1 points 1 month after final intervention). |
| Boggio *et al*. (2005) [48] | PD (*n* = 25) | TMS and fluoxetine | Participants allocated to either Group 1 (active rTMS and placebo fluoxetine) or Group 2 (sham rTMS and fluoxetine).  rTMS stimulation parameters: 10 daily sessions, 15Hz, 110% MT, applied to left DLPFC. | HAM-D, BDI and neuropsychological tests. | Mood improvements after both treatments (Group 1 BDI: ↓6.6 points at 2 week follow-up; ↓5.1 points at 8 week follow-up; Group 1 HAM-D: ↓8.2 points at 2 week follow-up, ↓6.6 points at 8 week follow-up).  Improved cognitive function observed after both treatments (Stroop test – coloured words: (Group 1 ↓2.8 points and ↓2.3 points at 2 and 8 week follow-up time points; Hooper (Group 1 ↑1.5 points and ↑2.1 points at 2 and 8 week follow-up time points) and Winconsin (preservative errors) (Group 1 ↓4.9 points at 2 and ↓7.3 points at 8 week follow-up time points)) after both treatments. |
| Boggio *et al.* (2006) [49] | PD (*n* = 18) | tDCS | Active or sham stimulation. Anode applied to either left DLPFC (F3) or primary motor cortex (M1) and cathode applied to contralateral right orbit.  Stimulation delivered at 1mA or 2mA for single 20 min session. | Three-back working memory task. | 2mA, but not 1mA, applied to the DLPFC improved accuracy (↑approx 20%) but not speed. |
| Cardoso *et al* (2008) [50] | PD (*n* = 21) | TMS and fluoxetine | Participants allocated to either Group 1 (active rTMS and placebo fluoxetine) or Group 2 (sham rTMS and fluoxetine).  rTMS stimulation parameters: three sessions per week, on alternating weekdays, for 12 sessions over four weeks. 50 trains of 15s delivered to left DLPFC (120% MT with 5Hz frequency). | HAM-D, BDI, MMSE, activities of daily living, (in addition to motor  outcome measures). | Improvements shown in BDI (Group 1: ↓8.5 points 4 weeks post-treatment), HAM-D (Group 1: ↓9.2 points 4 weeks post-treatment), MMSE (Group 1: ↑2.8 points 4 weeks post-treatment) and in daily living (as shown in Group 1 SE scores: ↓8.2 points). |
| Dragasevic *et al.* (2002) [51] | PD (*n* = 10) | TMS | Open-label study. Stimulation delivered to left and right prefrontal regions: MT+10% at 0.5Hz with 0.1ms pulse duration. Patients received 5 × 20 stimuli. Initial side of stimulation changed on every consecutive day with 5 minute interval between another side of stimulation. | HAM-D, BDI (and UPDRS). | Improvements in HAM-D (↓6.5 points at 2 hours follow-up, ↓7.4 points at 11 days follow-up and ↓7.2 points at 20 days follow-up), BDI (↓5.1 points at 2 hours follow-up, ↓7.4 points at 11 days follow-up and ↓7.2 points at 20 days follow-up). |
| Epstein *et al.* (2007) [52] | PD (*n* = 14) | TMS | Open-label study. 1000 rTMS pulses (10Hz) delivered to left DLPFC for 10 consecutive days (morning and afternoon) at 110% MT. | HAM-D (17 and 21 item), BDI, HAMA, BPRS, CGI, DRS, RBANS, BTA and UPDRS. | Improvements in 17-item HAM-D (↓6.2 points after completion of TMS and ↓8.5 points at 3-6 week follow-up) and 21-item HAM-D (↓7.8 points after completion of TMS and ↓10.8 points at 3-6 week follow-up), BDI (↓7.3 points after completion of TMS), HAMA (↓6 points after completion of TMS and ↓10.3 points at 3-6 week follow-up), DRS (↑5.6 points after completion of TMS; with improvements in DRS subscales) |
| Fregni *et al.* (2004) [53] | PD (*n* = 42) | TMS and fluoxetine | Participants allocated to either Group 1 (active rTMS and placebo fluoxetine) or Group 2 (sham rTMS and fluoxetine).  rTMS delivered to left DLPFC (40 trains of 5s pulses; 110% MT; 15Hz frequency) for 10 days during a two week period. | HAM-D, ADL, BDI, MMSE (and UPDRS). | Group 1: improvements in HAM-D (↓9.5 points two weeks after treatment) and BDI (↓8.1 points two weeks after treatment), with no significant differences between two-week and eight-week follow-up time points, comparable to Group 2. Group 1 showed improvement in ADL at eight-week follow-up (↑3 points; not observed in Group 2) and greater MMSE improvements than Group 2 at two-week follow-up (↑1.4 points) |
| Fregni *et al*. (2006) [54] | PD (*n* = 26) | TMS and fluoxetine | Aim of study was to examine effects of fluoxetine and rTMS upon regional cerebral blood flow. Participants allocated to either Group 1 (active rTMS and placebo fluoxetine) or Group 2 (sham rTMS and fluoxetine).  rTMS delivered to left prefrontal area (40 trains of 75 pulses over 5 secs, followed by 10 second intertrain interval; MT +10%; 15Hz frequency) for 10 sessions during a two week period. | HAM-D, ADL, BDI, MMSE (and UPDRS) | Improvements across both groups for BDI and HAM-D (indicated by analysis of variance showing a main effect of time at two-week and eight-week follow-up period but not a group × time interaction), with magnitude of improvement unspecified. |
| Furukawa *et al*. (2009) [55] | PD (*n* = 6) | TMS | Open-label study: 0.2Hz rTMS applied to frontal region (Fz) at 120% MT, 100 times once a week for a period of approximately 3 months (1,200 stimulations in total). | TMT-B, WCST, WAIS-R, SDS, FIM (and motor measure). | Improvements observed in TMT-B (↓157.7 seconds) and WCST scores (Categories Achieved ↑3.8 points; Perservative Errors of Nelson ↓6.3 points; Total Errors ↓15.3 points) at end of stimulation period. |
| Kormos *et al.* (2007) [56] | PD (*n* = 7) | TMS | Open-label study: 20Hz rTMS applied to left DLPFC at 80% MT; 2,000 pulses per treatment session (on for 2 secs; off for 28 secs, for 25 minutes). Total of 10 treatments were delivered in two weeks.  Responders (defined as individuals who showed a 50% decrease in HAM-D scores relative to baseline following an initial two weeks) entered an ‘Extension Phase’ and received four treatments once a week. | BDI-II, HAM-D, State and Trait Anxiety Scales. | Responders were followed up at 1 and 6 month time periods. Statistical analyses were not conducted:  Responders showed improvements in Trait Anxiety (↓9.2 points post-treatment; ↓9.9 points at 1 month and ↓7 points at 6-month follow-ups) and State Anxiety (↓10.2 points post-treatment; ↓3.9 points at 1 month and ↓4.1 points at 6-month follow-ups), BDI (magnitude of improvements not specified) and HAM-D (limited follow-up data available:↓15.8 points post-treatment; ↓16.8 points at 1 month and ↓15.2 points at 6-month follow-ups). |
| Pal *et al.* (2010) [57] | PD (*n* = 22) | TMS | 5Hz rTMS applied to left DLPFC at 90% MT; 600 pulses per treatment session for 10 days. | MMSE, MADRS, BDI, SE, TMT and Stroop test (in addition to UPDRS and Sleepiness). | Active stimulation group showed improvements in BDI (↓4 points at 1 day and 1 month follow-up periods), MADRS (↓1.5 points at 1 day and ↓3 points at 1 month follow-up period) and Stroop accuracy (↑11.5% at 1 day and ↑12.5% at 1 month follow-up period). |
| Sedlackova *et al.* (2009) [58] | PD (*n* = 10) | TMS | Crossover study: rTMS applied to left PMd and DLPFC (active stimulation sites) and to occipital cortex (control stimulation site). 10Hz stimulation (15 × 30 pulse trains at 100% MT separated by 10 sec intertrain interval; 1,350 stimuli in total). | Verbal Fluency test, TMT, Digit Span and reaction time tests. | No effects of active stimulation upon any measure. |
| Srovnalova *et al*. (2012) [59] | PD (*n* = 10) | TMS | Crossover study: rTMS applied to left and right DLPFC (active and sham stimulation). 25Hz stimulation applied at 80% RMT (10 sets × 30 pulses in two sessions; 600 pulses per day). | Tower of London task. | Improved performance after right DLPFC stimulation only (↓25 sec total problem-solving time). |
| Abbreviations: AMT: active motor threshold, BDI: Beck Depression Inventory, BDI-II: Beck Depression Inventory II, BPRS: Brief Psychiatric Rating Scale, BTA: Brief Test of Attention, CGI: Clinical Global Inventory; DLPFC: dorsolateral prefrontal cortex, DRS: Dementia Rating Scale; FIM: Functional Independence Measure, Hz: hertz, HAMA: Hamilton Anxiety Scale, HAM-D: Hamilton Rating Scale for Depression, iTBS: intermittent theta burst stimulation, mA: milliamps, MADRS: Montgomery-Astberg Depression Rating Scales; MT: motor threshold, MMSE: Mini-Mental State Examination, ms: millisecond, PD: Parkinson’s disease, PMd: dorsal premotor cortex, RBANS: Repeatable Battery for Assessment of Neuropsychological Status; RMT: resting motor threshold, rTMS: repetitive transcranial magnetic stimulation, SDS: Self-rating Depression Scale, SE: Schwab and England activities of daily living index , SF-12v2: Short Form Health Survey 12-item version 2, SRTT: Serial Reaction Time Task, tDCS: transcranial direct current stimulation, TMS: transcranial magnetic stimulation, TMT : Trail Making Test; TMT-B: Trail Making Test part B, UPDRS: Unified Parkinson’s Disease Rating Scale, WAIS-R: Wechsler Adult Intelligence Scale (Revised), WCST: Wisconsin card sorting test. | | | | | |
